# Improving the realism of deterministic multi-strain models: implications for modelling influenza A

**DOI:** 10.1098/rsif.2008.0333

**Published:** 2008-09-18

**Authors:** Pavlo Minayev, Neil Ferguson

**Affiliations:** Department of Infectious Disease Epidemiology, MRC Centre for Outbreak Analysis and Modelling, Imperial College Faculty of MedicineNorfolk Place, London W2 1PG, UK

**Keywords:** multi-strain model, population dynamics, cross-immunity, antigenic variation, strain diversity, influenza dynamics

## Abstract

Understanding the interaction between epidemiological and evolutionary dynamics for antigenically variable pathogens remains a challenge, particularly if analytical insight is wanted. In particular, while a variety of relatively complex simulation models have reproduced the evolutionary dynamics of influenza, simpler models have given less satisfying descriptions of the patterns seen in data. Here, we develop a set of relatively simple deterministic models of the transmission dynamics of multi-strain pathogens which give increased biological realism compared with past work. We allow the intensity of cross-immunity generated against one strain given exposure to a different strain to depend on the extent of genetic difference between the strains. We show that the dynamics of this model are determined by the interplay of parameters defining the cross-immune response function and can include fully symmetric equilibria, self-organized strain structures, regular periodic and chaotic regimes. We then extend the model by incorporating transient strain-transcending immunity that acts as a density-dependent mechanism to lower overall infection prevalence and thus pathogen diversity. We conclude that while some aspects of the evolution of influenza can be captured by deterministic models, overall, the description obtainable using a purely deterministic framework is unsatisfactory, implying that stochasticity of strain generation (via mutation) and extinction needs to be captured to appropriately capture influenza dynamics.

## 1. Introduction

Antigenically diverse pathogens are responsible for a major burden of disease in the modern world, with dengue, influenza A, meningitis and malaria being examples of infections that give rise to significant morbidity and mortality globally. Understanding how key factors (e.g. transmissibility, duration of infection, mutability, host population size) influence pathogen evolution and transmission dynamics is necessary for the rational design of intervention strategies to minimize the impact of infectious diseases.

The most important factor determining the fitness (i.e. transmission success) of antigenically variable pathogens is their ability to escape antigen-specific responses of hosts' immune systems. Cross-immunity is the mechanism used by hosts to combat antigenic escape—the ability of the immune system to mount a partially effective response against novel pathogen strains after previous exposure to older related strains. Ecologically, cross-immunity induces frequency-dependent inter-strain competition and can lead to the emergence of complex cyclical transmission dynamics (e.g. Dawes & Gog 2002; Gog & Swinton 2002; Grenfell *et al*. 2004; Recker *et al*. 2007). To formalize the quantification of inter-strain competition, it is convenient to use the notion of a ‘strain space’. Pathogen strains in such a space are represented as points, and the metric—which determines the distance between two points—serves as a measure of antigenic affinity between the corresponding strains (Smith *et al*. 1997, 1999). Using the concept of the strain space, cross-immunity can be incorporated into epidemiological models as a factor that depends on the antigenic distance between an attacking strain and strains to which a host possesses specific immunity, i.e. the host immune history.

The precise geometry of strain space has not yet been established for any existing pathogen, but various structures have been proposed. One of the simplest types, one-dimensional strain space, in which only nearest-neighbour interaction between strains located along a circle or line was considered, was used by Andreasen *et al.* in a phenomenological model of influenza evolution (Andreasen *et al*. 1996, 1997; Gomes *et al*. 2001). More complicated one- and two-dimensional spaces, in which strain interaction was described by a matrix, with cross-immunity being Gaussian in inter-strain distance, were used by Gog (Gog & Grenfell 2002) to describe dynamics and selection in multi-strain systems. These models can successfully reproduce certain aspects of real epidemic patterns such as a static discrete strain structure (DSS), increasing diversity and sequential strain replacement observed for dengue, HIV and influenza, respectively. However, they all have a significant common drawback that makes them somewhat uninformative—the biological basis of the assumed strain space is opaque. Furthermore, assuming strain space is one-dimensional means influenza-type evolution is almost pre-programmed, emerging as a soliton-type wave in strain space.

Even moving to a two-dimensional strain space, obtaining influenza evolution is far from assured: one has to assume that there is a fitness (i.e. transmissibility) gradient in one dimension in strain space, as done by Gog (Gog & Grenfell 2002). When we reproduced the Gog model without such a fitness gradient, circular waves in strain space were the dominant dynamical pattern seen. Biologically, the antigenic evolution of influenza needs a two- to three-dimensional space to be represented accurately (Smith *et al*. 2004), with perhaps over a dozen genetic loci in haemagglutinin contributing to its antigenicity (Bush *et al*. 1999).

A further challenge when developing compartmental models of antigenically variable pathogens is the increase in the dimensionality of state space with the number of strains modelled. Dimensionality increases geometrically with the number of strains for models that explicitly describe full immune histories (Andreasen *et al*. 1996, 1997; Gomes *et al*. 2001). Even for models where state space dimensionality increases linearly with the number of strains (Gupta *et al*. 1998; Gog & Swinton 2002), there are practical limits to the number of strains which can be modelled (of a few hundred to a few thousand strains). For pathogens with a relatively limited and static antigenic repertoire this is not necessarily an issue. However, for influenza, where the dominant pattern is the replacement of existing strains by new strains, upper bounds on the number of strains able to be modelled can limit the ability of models to capture evolutionary patterns. Some past work has made a virtue of these limits (Recker *et al*. 2007) and argued that the strain recycling predicted by models with very limited strain repertoires is a real feature of influenza dynamics. However, as we discuss later, the antigenic data to back this hypothesis are limited. While the potential antigenic diversity of influenza must in theory be limited, the large number of codons involved in determining antigenicity (Bush *et al*. 1999; Smith *et al*. 2004; Koelle *et al*. 2006) suggest that strain recycling, while theoretically possible, is probabilistically very unlikely.

Simulation offers a means for both substantially increasing the number of strains which can be modelled and more realistically capturing pathogen transmission and evolution, and such models have provided plausible descriptions of influenza evolution (Ferguson *et al*. 2003; Koelle *et al*. 2006). But these simulations are complex and computationally intensive, and thus isolating the key determinants of model behaviour can be difficult. To provide more insight into pathogen dynamics and evolution, simpler but at the same time more biologically realistic models need to be developed. Arguably the most promising approach to date, which combines relative simplicity with a genetic representation of antigenic diversity, was proposed by Gupta *et al.* (1998). Strains in this model are considered as sequences of loci, each of the loci can be occupied by one of a set of alleles. The distance between two strains is defined as the number of loci at which the alleles are different. An assumption is made that prior infection endows hosts with partial cross-immunity against infection with strains sharing alleles at the corresponding loci with an earlier strain. In the original model, a very simple cross-immune interaction between strains was assumed—the degree to which a host was protected against new infections was the same for all non-discordant strains.

Therefore in this paper, we focus on improving the biological realism of simple compartmental models of multi-strain pathogens. Specifically, we include three factors that may be critical in explaining the evolutionary dynamics of diseases such as influenza A (Ferguson *et al*. 2003): (i) rapid mutation (§2); (ii) more realistic phenotypic models of cross-immunity (§3); and (iii) transient strain-transcending immunity (§4). For each factor, we examine the impact of pathogen diversity and dynamics.

## 2. Model with no strain-transcending immunity

In this section we consider a deterministic model with no strain-transcending immunity. The model consists of a set of compartments representing parts of a population immune to and infectious with virus strains. These compartments can overlap, which means that the same parts of the population can be immune to or can be infected and become infectious with multiple strains simultaneously. Random mixing of the population is assumed, and immunity to a particular strain is lifelong. Strains are described as sequences of antigens consisting of *N*_L_ loci, each of which can be occupied by one of *N*_A_ alleles. Thus, the total possible number of strains in the model is $N_{\text{S}}=N_{\text{A}}^{N_{\text{L}}}$.

The dynamics of the part of the population susceptible to a strain *i* are given by

(2.1) $$\frac{\text{d}s_{i}}{\text{d}t}=-(\lambda_{i}+\mu )s_{i}+\mu .$$

In this equation, *λ*_*i*_ is the force of infection defined as *λ*_*i*_=*βy*_*i*_, where *β* is the transmission coefficient, which we assume to be the same for all strains; *y*_*i*_ is the proportion of infectious with strain *i*; and *μ* is the natural birth and death rate (we suppose birth and death processes in the population to be at equilibrium).

To quantitatively characterize affinity of strains, we use the Hamming distance, i.e. we define the inter-strain distance as the number of loci occupied by different alleles. This value can take on discrete values from the set [1, 2, …, *N*_L_].

We also define a set of additional compartments, $s_{i}^{\left(k\right)}$ (*k*=1, …, (*N*_L_−1)), which represent the proportion susceptible to any strain *j* that shares alleles at the corresponding loci with strain *i* but has not more than *k* alleles different from *i*. From the definition, it is clear that the proportion of susceptible to strains sharing alleles with strain *i* but having exactly *k* distinct alleles is $\left(s_{i}^{\left(k\right)}-s_{i}^{\left(k-1\right)}\right)$. We expand the definition of $s_{i}^{\left(k\right)}$ and put $s_{i}^{\left(N_{\text{L}}\right)}=0$ and $s_{i}^{\left(0\right)}=s_{i}$. Dynamics of $s_{i}^{\left(k\right)}$, where *k*=1, …, (*N*_L_−1), is described by the equations

(2.2) $$\frac{\text{d}s_{i}^{\left(k\right)}}{\text{d}t}=\mu (1-s_{i}^{\left(k\right)})-s_{i}^{\left(k\right)}\sum_{j:d_{ij}\leq k}\lambda_{j}.$$

Here *d*_*ij*_ is the distance between strains *i* and *j*, and the summation is done over all strains, the distances between which are less than or equal to *k*.

Cross-immunity affects strain dynamics profoundly, and gives rise to several distinct dynamical regimes (Gupta *et al*. 1998; Ferguson & Andreasen 2002). Here we assume that the degree to which a host immune to strain *i* is protected against infection with strain *j* is defined by the number of alleles shared by the two strains. More specifically, we define *d* as the number of loci at which *i* and *j* differ, and a cross-immunity function *γ*(*d*), a non-increasing function of *d*. Those who are immune to strain *i* and have been exposed to strain *j* get infected with probability (1−*γ*(*d*)). Thus, *γ*(*d*)=1 corresponds to complete protection, while *γ*(*d*)=0 implies complete susceptibility. This definition extends the two-level cross-immunity model used by Recker & Gupta (2005).

Another important factor is mutation that helps virus escape pre-existing immunity. In the model that we study, mutation is characterized at the level of individual hosts (rather than virions), with the parameter *m* representing the probability per unit time that the strain type of someone currently infected changes due to mutation. We assume that, as a result of mutation, hosts acquire infectiousness with a new strain and that mutation can only generate strains one allele different from the current strain (i.e. one point mutations).

We thus obtain the equations for the temporal evolution of the proportion of people infectious with strains *i* (*i*=1, …, *N*_S_)

(2.3) $$\frac{\text{d}y_{i}}{\text{d}t}=\lambda_{i}\sum_{j=0,\ldots ,(N_{\text{L}}-1)}(s_{i}^{\left(j\right)}-s_{i}^{\left(j+1\right)})(1-\gamma (j+1))-\sigma y_{i}+\sum_{j=1,\ldots ,N_{\text{S}},j\neq i}m_{ji}(y_{j}-y_{i}).$$

In this equation, *σ* is the rate of loss of infectiousness of the host (so 1/*σ* is the mean duration of infectiousness). The last term describes mutation, with *m*_*ji*_=*m*_*ij*_=*m* if the distance between strains *i* and *j*, *d*_*ij*_, is 1 and *m*_*ji*_=0 otherwise. We consider infections for which the recovery rate is much higher than the natural death rate of hosts, so the value of *σ* is mainly determined by the recovery rate.

The number of differential equations describing our model is $C=N_{\text{A}}^{N_{\text{L}}}(N_{\text{L}}+1)$. The dimensionality of the model scales exponentially with the number of loci and as a power function with the number of alleles.

## 3. Model dynamics

We now numerically investigate the dynamical properties of the model described in §2. In this section, we assume an antigenic genotype of five loci and three alleles, giving a total possible number of different strains of *N*_S_=243. This assumption is relaxed later in the paper. The mutation rate, *m*, for the virus is assumed to be 10^−4^/year (though it should be noted that model dynamics are almost identical for 10^−5^<*m*<10^−3^). We assume a transmission coefficient of *β*=100/year and a recovery rate of *σ=*50/year (giving a basic reproduction number, *R*_0_, of 2). The birth–death rate, *μ*, is 0.014/year, which corresponds to a life expectancy of approximately 70 years.

To characterize the dynamics of the model, we use bifurcation diagrams built by plotting (locally) maximum values of *Z*_*i*_ (proportion immune to strain *i*) for all *i*=1, …, *N*_S_, after the phase trajectory of the system arrives at an attractor. To achieve this, we discard the first 10 000 year period of each solution, and analyse the subsequent 5000 years. At *t*=0, we start the system with two strains that differ at two loci and have slightly different prevalences.

First, we consider the case studied in Gupta *et al*. (1998) and Ferguson & Andreasen (2002) where the probability for a person exposed to one strain to be infected with another one is the same for all non-discordant strains regardless the genetic distance between them. This model has a single cross-immunity parameter, *b*. The function *γ*(*d*) takes the form *γ*(*d*)=*b*, where *b* is constant for 1≤*d*<*N*_L_ and *γ*(*d*)=0 for *d*=*N*_L_ (this property means that there is no cross protection against discordant strains).

The model reveals three distinct types of dynamical behaviour (figure 1; Gupta *et al*. 1998). The first dynamical regime, no strain structure (NSS), corresponding to a symmetrically fixed point where all strains coexist, is found for 0≤*b*≤*b*_L_, where *b*_L_=0.36 for our choice of parameters. As cross-immunity increases above the *b*_L_ threshold, competition between strains starts to structure the pathogen population and leads to chaotic/cyclical strain structure (CSS), with complex quasi-periodic and chaotic dynamics in the range *b*_L_<*b*≤*b*_U_, where *b*_U_=0.71 for our choice of parameters. Both the amplitude and period of epidemic oscillations grow as the value of *b* approaches *b*_U_. Further increase of the degree of cross-immunity, above the value of *b*_U_, leads to an abrupt transition to a new equilibrium—DSS. In the *b*_U_<*b*≤1 region, the system structures itself into sets of discordant strains, where all members of a single discordant set share no common alleles with each other. One discordant set dominates, with all members being at a high prevalence, while all other strains have a very low prevalence and are only maintained by the ongoing mutation.

Assuming the same level of cross-immunity against all non-discordant strains allows analytical derivation of the equilibrium points of the model and their stability (Ferguson & Andreasen 2002). However, the model is biologically simplistic as the degree to which a person exposed to one strain is protected against another should be dependent on how phenotypically or genetically close these strains are. To make the model more biologically realistic and yet retain some simplicity, we use the following two-parameter form of *γ*(*d*):

(3.1) $$\gamma (d)=\{\begin{array}{ll} 1, & \text{if}\;d=0\\ b\cdot [1-(d-1)a], & \text{if}\;d=1,2,\ldots ,\leq (1/a+1)<N_{\text{L}}\\ 0, & \text{otherwise}, \end{array}$$

where *a* and *b* are constants that characterize, respectively, the slope of the function, i.e. how fast the cross-immunity protection decays with the distance between strains, and the maximal value of cross-immunity attained when a new strain has only a single allele that differs from previous strains. Note that *a*=0 corresponds to cross-immunity that does not decay with genetic distance, and *a*=1 corresponds to cross-immunity *b* at distance 1, and zero cross-immunity for any larger genetic distance. An example of this function is plotted in figure 2.

In this case, the dynamics of the system can be more complicated and are determined by the interplay between both of these cross-immunity parameters. This is shown in figure 3*a*,*b* where we map model dynamical behaviour as a function of the parameters *a* and *b* for two model variants differing in the size of their antigenic ‘genome’. The same three dynamical regimes are seen as for the simpler model presented earlier (corresponding to the *a*=0 line in figure 3*a*). It is interesting to note that for this more general cross-immunity model, the region of parameter space supporting DSS dynamics is very small, corresponding to very intense cross-immunity between all strains. Transition from DSS to chaos always occurs through rather narrow zones of long-periodic regimes via Andronov–Hopf bifurcations. Within the CSS region, there is a huge variety of dynamical behaviour. Bifurcation plots give insight into this, and figure 4*a–d* show bifurcation behaviour as a function of *a* for four fixed values of *b*. For moderate maximal values of cross-immunity, *b*_L_<≈*b*<≈*b*_U_, a number of different types of the system dynamical behaviour can be distinguished as *a* changes (figure 4*a*): small amplitude chaotic epidemic oscillations at low *a* (see the interval 0≤*a*<0.15), then the fully symmetric equilibrium (the smooth curve in the interval 0.14≤*a*<0.38), then quasi-periodic oscillations (0.38≤*a*<0.62), giving way to chaotic behaviour (the rest of the *a*-axis). A few apparent trends can be noted: as the cross-immune response becomes narrower (i.e. as *a* increases and *γ*(*d*) decays more rapidly), strain infection prevalence grows together with the amplitude of epidemic oscillations in chaotic regimes, while the period of oscillations falls.

At larger maximal values of cross-immune response, *b*<≈*b*_U_, the dynamics of the system undergo qualitative changes in the second chaotic region of higher *a* values (*a*>0.27 in figure 4*b*). Larger ‘windows’ of limit cycle behaviour begin to appear in the chaotic region (figure 4*b*, 0.5≤*a*<0.68). These regions correspond to long period solutions, an example of which is shown in figure 5. The long-period limit cycle regions in the bifurcation graph grow as *b* approaches the second threshold value, *b*_U_, the value at which cross-immunity is strong enough to structure the population into discordant strain sets. The chaotic regimes are largely replaced by limit cycles (figure 4*c*, 0.3≤*a*<0.72) and point attractors (figure 4*c*, 0.72≤*a*<1). Note that the point attractors obtained are not completely symmetric (i.e. equilibrium strain prevalences may differ).

Once *b*>*b*_U_, discrete structure is seen for small *a*, though the equilibria seen in past work (Gupta *et al*. 1998) are only stable when *a* is very small (*a*<0.03 in figure 4*d*). As *a* increases, an Andronov–Hopf bifurcation occurs and a long-period limit cycle appears in place of the fixed point (the region 0.03<*a*<0.05 in figure 4*d*). An example of this behaviour is shown in figure 6*a*. As a result of the bifurcation, strain prevalences occasionally change abruptly, but strains still remain structured into discordant sets. Further increases in *a* lead to this periodic behaviour destabilizing and being replaced by chaotic dynamics (0.05≤*a*<0.35) with DSS breaking down (see figure 6*b* for an example). The region 0.35≤*a*<1.0 has a complex alternating structure corresponding to different dynamical regimes; small changes in *a* can cause switching between fixed points, long period, and quasi-periodic solutions. Examples of these solutions are presented in figure 6*c*,*d*.

## 4. Including strain-transcending immunity

The model considered in §§2 and 3 does not incorporate short-lived strain-transcending immunity of the type that simulation studies of viral evolution have shown to potentially play a key role in explaining the limited strain diversity and cluster dynamics of human influenza evolution (Ferguson *et al*. 2003). Here, we extend the system of equations (2.1)–(2.3) from §2 to include such a short-lived non-specific immune response into our simpler compartmental model. We show that such immunity (which biologically might be mediated by T-cell responses against conserved epitopes) leads to a reduction of strain diversity and qualitative and quantitative changes in model dynamics.

We suppose that upon infection a host is removed from the susceptible subpopulation and enters a state where they are temporarily immune to all strains (as well as gaining permanent immunity to the strain they were exposed to). Defining that the rate of returning from this completely immune state as *γ*, we introduce the following equations to describe dynamics of the temporarily immune population:

(4.1) $$\frac{\text{d}r_{i}^{\left(k\right)}}{\text{d}t}=s_{i}^{\left(k\right)}\sum_{j:d_{ij}>k}\lambda_{j}-(\gamma +\mu )r_{i}^{\left(k\right)}.$$

Equations (2.1) and (2.3) remain unchanged, whereas equation (2.2) is superseded by

(4.2) $$\frac{\text{d}s_{i}^{\left(k\right)}}{\text{d}t}=\mu (1-s_{i}^{\left(k\right)})-s_{i}^{\left(k\right)}\sum_{j:\forall d_{ij}}\lambda_{j}+\gamma r_{i}^{\left(k\right)}.$$

In both (4.1) and (4.2), indexes *i*=1, …, *N*_S_ and *k*=1, …, *N*_L_−1. Thus, the number of differential equations describing the model is $C=2N_{\text{L}}N_{\text{A}}^{N_{\text{L}}}$ and, as for the simpler model considered earlier, model dimensionality scales exponentially with the number of loci and as a power of the number of alleles.

To compare the dynamics of models with and without strain-transcending immunity, we map the dynamical behaviour as a function of *a* and *b* as before, and also plot weighted strain diversity (i.e. the average prevalence-weighted number of co-circulating strains) in the same phase plane (figure 7*a–d*).

Including strain-transcending immunity qualitatively changes system dynamics at higher values of cross-immunity (*b*>0.45). Short-lived immunity leads to self-organized strain structure (DSS area) disappearing and its replacement by regular long-period limit cycles. Similarly the NSS regions (where all strains have equal stable prevalence) also vanish, being replaced by chaotic dynamics.

The prevalence-weighted number of circulating strains ranges from 5 to 35 for the periodic and chaotic regimes at *b*>0.45, when compared with 15–40 in the absence of non-specific immunity.

An important aspect of influenza A evolution any model needs to reproduce is the observed 3–5 year interval between emergence of antigenically distinct strain clusters (Smith *et al*. 2004). Since we do not include seasonal forcing in this model, the frequency of emergence of antigenically novel strains is just given by the frequency of epidemics. Models with strain-transcending transient immunity have a much larger region of parameter space where the mean period between strain emergence is comparable with influenza data. As shown in figure 8*a*,*b*, strain-transcending immunity substantially shortens the mean period between epidemics. Model without this transient immunity typically gives epidemic oscillations with periods from a few decades to a few hundred years (figure 8*a*) for the majority of combinations of cross-immunity parameters *a* and *b*>0.45, whereas the period falls to under 2–5 years for a wide area of parameter space when transient immunity is added (figure 8*b*). Example time series are shown in figure 9*a*,*b*, which highlight how different from influenza the dynamics of the model without transient non-specific immunity can be.

Average strain diversity grows with the complexity of the virus genotype. The results presented in figure 10*a* are in agreement with those in Recker *et al*. (2007) for the *δ*=0 case of the model studied in that paper (*b* is the analogue of the immunity parameter *γ* in Recker *et al*. 2007): lower values of *b* (<≈0.4, …, 0.62) correspond to symmetric equilibria with all strains coexisting—in this case the number of strains present of course increases exponentially with the number of loci. At higher values of *b* cross-immunity reduces diversity overall, and in the extreme case of *a*=0 (figure 10*a*) this results in average numbers of strains increasing sub-linearly with the number of loci for *b* approaching 1 (in the absence of mutation, the number of strains equals the number of alleles for *b*=1 and *a*=0, but mutation complicates this simple picture). For larger values of *a* (figure 10*b*), we recover close to the same exponential dependence of the number of strains on the number of loci, albeit diversity overall still reduces with increasing *b*. Transient non-specific immunity is seen to have a noticeable effect on strain diversity for more complex genotypes at values of *a*>0.5 (figure 10*d*), i.e. where three or fewer allele substitutions are sufficient to escape prior immunity. For *a*=0.6 and 0.75<*b*<1 there is approximately a two- to threefold difference in strain diversity between the models with and without transient immunity (comparing figure 10*b* with *d*). For smaller values of *a*, the difference in strain diversity is less marked, though still significant for five or more loci.

Thus overall, including transient strain-transcending immunity improves the ability of models with genetic strain spaces to produce dynamics resembling that seen for influenza A in humans, since it gives a generally lower mean strain diversity and much more frequent emergence of new strains across a much wider area of the cross-immunity parameter space.

## 5. Conclusions

In this work, we have studied a class of multi-strain deterministic epidemic models in which cross-immunity varies with the genetic distance between strains. At low maximal values of cross-immunity (*b*<≈0.4) dynamics of the model are characterized by fully symmetric equilibria (all strains present), low amplitude periodic dynamics and chaos. At intermediate and high maximum values of maximal cross-immunity *b*≈>0.4, dynamics are more diverse, with parameter space including ‘windows’ of periodic dynamics interleaved with chaos, as well as boundary equilibria corresponding to the self-organization of virus strains into pairwise discordant sets. Such discordant set boundary equilibria are unstable to variation in the shape of the cross-immunity function *γ*(*d*): as this function gets steeper (meaning antigenic escape can occur with increasingly few genetic changes), first the boundary equilibria change to a limit cycle via an Andronov–Hopf bifurcation, and then a transition to a chaotic attractor occurs. These changes reflect a weakening of intense inter-strain competition seen for flat *γ*(*d*).

In very general terms, the three broad classes of behaviour exhibited by the model we have developed in this paper—namely all strains coexisting, static strain structure (finite subset coexisting) and oscillatory dynamics—are shared by many multi-strain models (Andreasen *et al*. 1997; Gupta *et al*. 1998; Gomes *et al*. 2001; Dawes & Gog 2002; Gog & Swinton 2002; Recker *et al*. 2007). However, this observation neglects the very significant differences in the type of oscillatory dynamics seen in different models. In addition, while selection for immune escape means that deterministic multi-strain models can often generate travelling waves in strain space if the dimensionality of strain space is low (Andreasen *et al*. 1997; Gomes *et al*. 2001; Gog & Grenfell 2002), behaviour is more complex in higher dimensional strain spaces and is more typically characterized by increasing diversification until strain space has been completely explored.

Given antigenic and genetic analyses of influenza evolution (Bush *et al*. 1999; Smith *et al*. 2004; Koelle *et al*. 2006) indicate that multiple (greater than 10) codons are involved in determining HA antigenicity, it would appear that models assuming one-dimensional strain spaces—while they may phenomenologically reproduce flu-like evolutionary dynamics—do not give a mechanistic explanation of what limits the antigenic diversity of influenza at each point in time. Other recent work has proposed that influenza A evolution is characterized by strain recycling (Recker *et al*. 2007), but this hypothesis relies on the assumption that antigenic mapping studies (Smith *et al*. 2004) have relied on only measuring cross-immunity (haemagglutinin inhibition, HI) between strains that circulated in neighbouring years—thus evidence of recycling was missed. In fact, HI tests between strains spanning the entire evolutionary history of H3N2 have now been completed, and no evidence of antigen recycling has been found (D. J. Smith 2005–2006, personal communication). Furthermore, figure 10*a* shows that limited diversity is only readily obtained from ‘genetic’ strain space models of the type used here and in past work (Gupta *et al*. 1998; Recker & Gupta 2005; Recker *et al*. 2007) when the number of antigenic loci is relatively low. In addition, once one allows for cross-immunity decaying with genetic distance, diversity increases still further (figure 10*b*), to levels substantially above what is seen in actual influenza A evolution.

Given these issues, and motivated by prior simulation studies (Ferguson *et al*. 2003), we therefore extended the model to incorporate transient strain-transcending immunity, which acts as a global mechanism to reduce infection prevalence and thus strain diversity. When strain-specific cross-immunity is relatively strong (*b*>≈0.45), the addition of strain-transcending immunity results in major changes in model dynamics: discordant strain structure is replaced by long-period limit cycles and chaotic oscillations emerge in place of symmetric and non-symmetric equilibria. Strain-transcending immunity also leads to a general decrease in the mean period between epidemics, higher amplitudes of epidemic oscillations, and results, as expected, in reductions in strain diversity. Overall, model dynamics are more reminiscent of influenza than in the absence of transient immunity, but diversity still remains rather high, especially as the number of antigenic loci increases to numbers realistic for influenza (i.e. 10 or more).

High strain diversity is a common drawback of all deterministic models, as in the presence of mutation, all strains are constantly present, with no strain truly going extinct. In ecological terms, the carrying capacity of the population is indefinitely large. In population genetic terms, the effective population size is infinite. We conclude that while deterministic models with a high-dimensional genetic strain space can reproduce some aspects of influenza dynamics, a full explanation is still lacking. Therefore, the theoretical challenge that we explore in the companion paper is how to introduce stochasticity and extinction into the relatively simple modelling framework developed in this paper without the use of a full microsimulation approach.

## Figures and Tables

**Figure 1:**
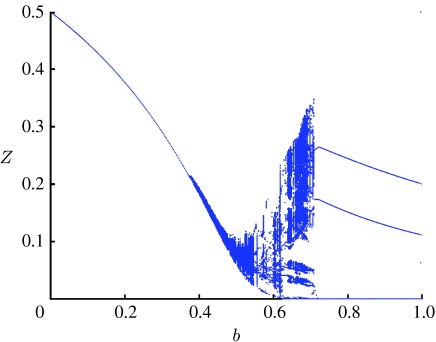
Bifurcation diagram plotting local maxima (taken from the last 5000 years of a 15 000 year solution of the model) of the proportion immune to each strain, *Z*_*i*_, as a function of the cross-immunity parameter *b*. The plot illustrates the different types of dynamical behaviour of the model obtained as *b* varies. *R*_0_=2 and the recovery rate *σ*=50/year.

**Figure 2:**
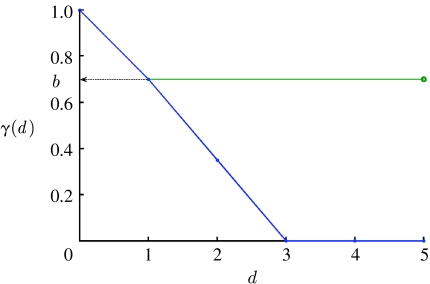
Example of the functional form used to describe the decline in cross-immunity, *γ*(*d*), with genetic distance, *d* (*a*=0.5, blue curve). *a*=0 corresponds to a flat function not changing with genetic distance (green curve).

**Figure 3:**
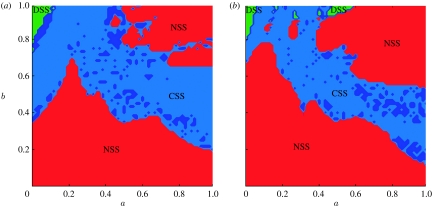
Dynamical maps for the models with (*a*) five locus three allele genotype and (*b*) eight locus two allele genotype. Parameter *a* (OX axis) characterizes decay of cross-immune response with genetic distance and *b* (OY axis) is the maximum cross-immunity level. In red areas, marked as NSS (no strain structure), dynamics converge to a point attractor with all strains present at constant prevalences. Light blue marks areas of CSS where quasi-periodic oscillations of prevalence occur. Dark blue indicates the small regions of pure periodic behaviour. Light green zones indicate DSS, where cross-immunity causes the system to self-organize into discordant (antigenically non-overlapping) sets of strains at constant prevalence.

**Figure 4:**
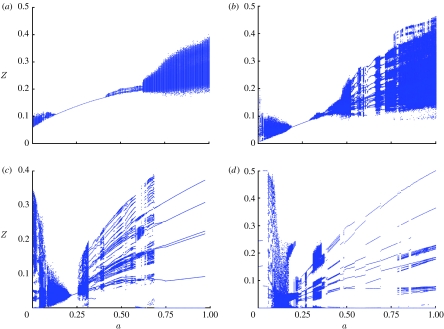
Bifurcation diagrams that characterize the model dynamics depending on the cross-immunity parameter *a*. Genotype consists of five loci and three alleles. *R*_0_=2, *σ*=50 yr^−1^, and *b* is (*a*) *b*=0.5, (*b*) *b*=0.6, (*c*) *b*=0.7, (*d*) *b*=0.8.

**Figure 5:**
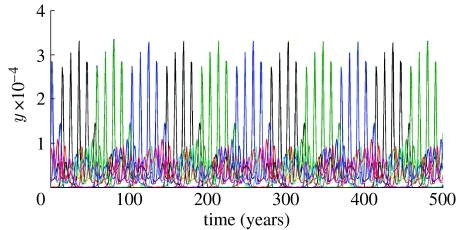
Strain infection prevalence for *a*=0.62 and *b*=0.6, taken from the region of long-period limit cycles figure 4*b*. *R*_0_=2 and *σ*=50/year.

**Figure 6:**
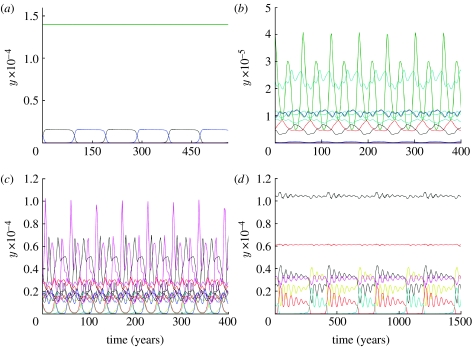
Time series of strain prevalence for example long-period solutions of the model for values of *a* above the Andronov–Hopf bifurcation at *a*=0.03 (*b*=0.8, *R*_0_=2, *σ*=50/year): (*a*) *a*=0.038; (*b*) *a*=0.28; (*c*) *a*=0.44; (*d*) *a*=0.81.

**Figure 7:**
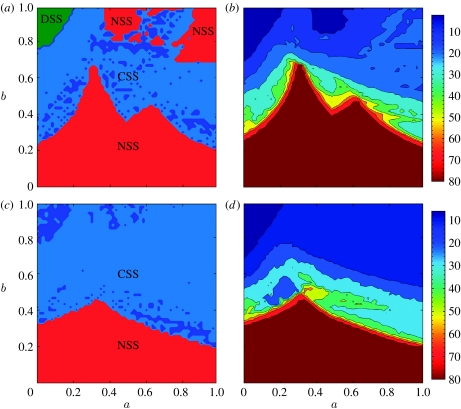
(*a*) Dynamical map for model with no strain-transcending immunity, with an antigenic genotype of four loci and three alleles. Dynamical regimes labelled as figure 3*a*; (*b*) as (*a*) but showing average prevalence-weighted number of strains; (*c*) as (*a*) but for model with strain-transcending immunity, assuming *γ*=3/year (i.e. four month duration of strain-transcending immunity); (*d*) as (*c*) but showing average prevalence-weighted number of strains.

**Figure 8:**
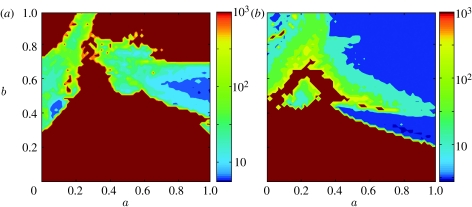
Map showing mean period between peaks in infection incidence (reflecting the frequency with which new strains emerge) as a function of the cross-immunity parameters *a* and *b*, for models with four locus three allele antigenic genotype and (*a*) no strain-transcending immunity; (*b*) with strain-transcending immunity (other model parameters are the same as in figure 7). Colour scale shows period in years on logarithmic scale.

**Figure 9:**
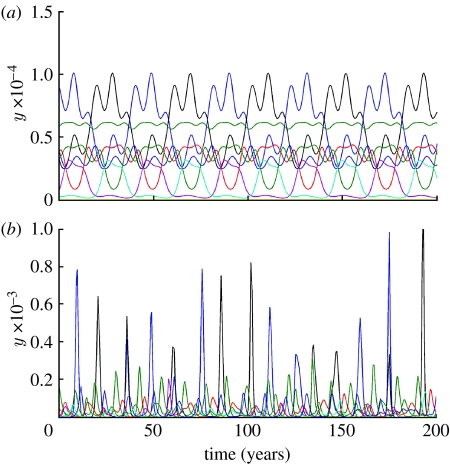
Sample time series of infection prevalence showing difference in dynamics and mean periods of epidemics for models (*a*) without strain-transcending immunity; (*b*) with strain-transcending immunity. Antigenic genotype consists of four loci and three alleles, cross-immunity parameters are *a*=0.7 and *b*=0.8 in both cases and other parameters as figure 7.

**Figure 10:**
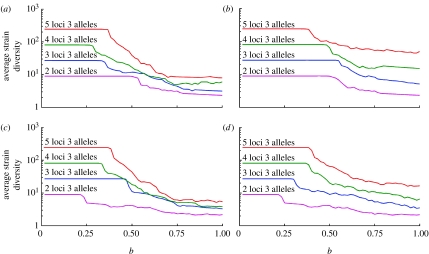
Dependence of average prevalence-weighted strain diversity on the cross-immunity parameter *b* for (*a*) *a*=0 and no strain-transcending immunity, (*b*) *a*=0.6 and no strain-transcending immunity, (*c*) *a*=0 with strain-transcending immunity and (*d*) *a*=0.6 with strain-transcending immunity (other main model parameters are the same as in figure 7).
